# Is It Possible to “Find Space for Mental Health” in Young People? Effectiveness of a School-Based Mental Health Literacy Promotion Program

**DOI:** 10.3390/ijerph15071426

**Published:** 2018-07-06

**Authors:** Luísa Campos, Pedro Dias, Ana Duarte, Elisa Veiga, Cláudia Camila Dias, Filipa Palha

**Affiliations:** 1 Faculty of Education and Psychology, Universidade Católica Portuguesa, Rua Diogo Botelho, 1327, 4169-005 Porto, Portugal; pdias@porto.ucp.pt (P.D.); aiduarte@porto.ucp.pt (A.D.); eveiga@porto.ucp.pt (E.V.); fpalha@porto.ucp.pt (F.P.); 2 Research Centre for Human Development, Rua Diogo Botelho, 1327, 4169-005 Porto, Portugal; 3 Department of Community Medicine, Information and Health Decision Sciences, Faculty of Medicine of the University of Porto, 4200-319 Porto, Portugal; camila@med.up.pt; 4 CINTESIS—Center for Health Technology and Services Research, Alameda Prof. Hernâni Monteiro, 4200-319 Porto, Portugal; 5 ENCONTRAR+SE—Association for the Promotion of Mental Health, Rua Professor Melo Adrião 106, 4100-340 Porto, Portugal

**Keywords:** mental health literacy, effectiveness, young people, promotion

## Abstract

Lack of knowledge regarding, and the stigma associated with, mental disorders have been identified as major obstacles for the promotion of mental health and early intervention. The present study aimed to evaluate the effectiveness of a school-based intervention program focused on the promotion of mental health literacy (MHL) in young people (“Finding Space for Mental Health”). A sample of 543 students (22 classes), aged between 12 and 14 years old, participated in the study. Each class of students was randomly assigned to the control group (CG; *n* = 284; 11 classes) or the experimental group (EG; *n* = 259; 11 classes). MHL was assessed using the Mental Health Literacy questionnaire (MHLq), which is comprised of three dimensions—Knowledge/Stereotypes, First Aid Skills and Help Seeking, and Self-Help Strategies. The scores on these dimensions can also be combined to give an overall or total score. Participants from the EG attended the MHL promotion program (two sessions, 90 min each) delivered at one-week intervals. Sessions followed an interactive methodology, using group dynamics, music, and videos adapted to the target group. All participants responded to the MHLq at three points in time: pre-intervention assessment (one week prior to the intervention), post-intervention assessment (one week after the intervention) and follow-up assessment (six months after the intervention). The intervention effectiveness and the differential impact of sociodemographic variables on the effectiveness of the program were studied using a Generalized Estimation Equation (GEE). Results revealed that participants from the EG demonstrated, on average, significantly higher improvement in MHL from pre-intervention to follow-up when compared to participants from the CG. Different sociodemographic variables affected the effectiveness of the program on distinct dimensions of the MHLq. Overall, “Finding Space for Mental Health” showed efficacy as a short-term promotion program for improving MHL in schools.

## 1. Introduction

In recent years, the literature has highlighted a growing concern about the number of children and youth who are experiencing mental health problems [1,2]. From a preventative perspective, it has become evident that there is an urgent need to intervene as early as possible in order to promote positive mental health and well-being [3,4]. This evidence is reinforced, in particular, by the fact that most mental disorders develop during youth, and it is estimated that about half of all cases of diagnosed mental disorders in adulthood started by the age of 14 [5,6,7,8].

With these factors in mind, several countries are currently investing not only in structures for the treatment of mental disorders, but also in the promotion of mental health and prevention of mental health problems both in at-risk groups and in the general population [9]. One of the five priority areas addressed by the EU’s Joint Action for Mental Health and Well-Being [10], launched in 2013, was the promotion of mental health in schools. This effort in treatment, prevention and promotion faces important challenges which are well reported in the literature—including the lack of adequate knowledge of mental health issues (mental health literacy) and the stigma associated with mental health problems [4,11].

The term mental health literacy was first introduced in 1997 by Jorm and colleagues and defined as “knowledge and beliefs about mental disorders which aid their recognition, management and prevention” [12]. It is known that the general population, and in particular young people, have low levels of mental health literacy (MHL) (e.g., [13]): they have difficulties in identifying mental disorders and their underlying causes, risk factors, and associated protective factors, and can develop incorrect beliefs about the effectiveness of therapeutic interventions, often resulting in a decrease in the likelihood of seeking help [14,15]. Additionally, the stigma associated with mental health problems becomes apparent to people at an early age [16]. However, the attitudes of young people are malleable and can be changed more easily than those of adults [17], and this therefore represents an important opportunity to invest in the promotion of mental health literacy at this age.

According to Wright, Jorm, Harris and McGorry [18], the recognition of mental health problems and associated psychological disorders is the most predictive factor for whether a young person seeks help. This predictor is of particular relevance because of the need to address the serious consequences to mental health that the delay in seeking help may entail [15,19,20].

Researchers from several countries have created programs for the promotion of mental health for young people, most of which were developed in a school context, whose main objective is to promote improved mental health by increasing MHL and reducing the stigma associated with mental health problems [21]. Overall, evaluation studies of these programs have demonstrated their effectiveness in improving MHL levels, although limitations have been identified in most studies—absence of focus groups and pilot studies with the target group; weak internal validity, causal assessment, and quality of evidence; and lack of long term follow-up, thus highlighting the need to develop new work that meets more stringent design criteria (e.g., [22,23,24]).

Evidence also suggests that some specific variables may influence the development of MHL, such as gender and prior contact with people who have mental health problems. With regards to gender, results from different studies suggest that young women have higher levels of MHL (e.g., [25,26,27,28]), presenting with more positive attitudes and less stereotypes in relation to people with mental disorders, when compared with young men [26,28,29]. In a study developed by Pinfold et al. [11] it was pointed out that, after an intervention to promote MHL, female participants obtained higher gains in MHL when compared to male participants.

Additionally, higher levels of MHL are consistently reported in people who refer knowing someone with a mental health problem (e.g., [30,31,32]). This prior contact seems to increase the impact of MHL promotion interventions with young people [11].

This article presents results regarding the effectiveness of a school-based intervention program focused on the promotion of mental health literacy in a group of young people, developed in the project “Finding Space for Mental Health” [21,33]. The specific objectives of this research were: (1) to evaluate the effectiveness of the intervention in the promotion of MHL; and (2) to analyze the impact of the variables of gender and previous contact with people with mental health problems, as well as to explore the potential impact of other sociodemographic variables of the participants, including year of schooling and type of school in the intervention results. We hypothesized that: (1) students who participate in the mental health literacy promotion program would display significant increases in MHL after the intervention, maintaining them at follow-up, compared to students who did not participate; (2) girls and students who have had previous contact with someone with a mental health problem would show higher increase in MHL after the program, compared to boys and to students who didn’t have previous contact with mental health problems.

## 2. Materials and Methods

### 2.1. Participants

Five-hundred and forty-three youngsters from 22 school classes were randomly divided into two groups—11 classes in the experimental group (EG: *n* = 259, 48%) and 11 classes in the control group (CG: *n* = 284, 52%), before pre-intervention assessment. The participants were aged between 12–14 years (mean age = 13.04, SD = 0.79), and were attending the third cycle of their basic education (26% in the seventh year, 37% in the eighth year, 37% in the ninth year) at one of eight schools in northern Portugal. The majority of the participants were male (52%) and attended state-funded schools (74%) (The English definition of public vs state-funded schools was used in this work—a public school is a school in which students have to pay to study and a state-funded school is a school that is free for students as the government pays for their study).

Concerning the professional situation of the parents, 86% were employed.

With regards to contact with mental health problems, 236 (44%) said they knew someone with a mental health problem, 161 (30%) reported not knowing anyone, and 139 (26%) did not know. As to the degree of proximity to a person with a mental health problem, 103 (42%) of the young people reported knowing a relative, 98 (39%) pointed out a friend, 8 (3%) said it was themselves, 37 (15%) pointed out another person, and 2 (1%) identified that several people with a mental health problem were known to them.

When questioned about the mental health problems experienced by the people they identified, 14% said they did not know. From the answers given by those who did know, 29 distinct problems were reported, some of them corresponding to mental disorders (e.g., “Depression”, *n* = 41), neurological problems (e.g., “Stroke”, *n* = 3) or other physical health problems (e.g., “Heart Problem”, *n* = 1).

Finally, there were no significant associations between the variables of the groups (experimental vs. control) and the sociodemographic variables, except for the type of school (public school vs. state-funded school) (Table 1).

The number of participants after the intervention decreased to 239 (EG) and 263 (CG), and during the follow-up assessment further decreased to 211 (EG) and 176 (CG), respectively. This was due to the school absence of some students in post-intervention assessment and to school year transition by students between post-intervention and follow-up (some students were transferred to a new school). No students who stayed in the same class/school refused to participate in the follow-up assessment.

### 2.2. Intervention

The intervention program, named “Finding Space” was composed of two sessions, lasting 90 min each and delivered at one-week intervals in the students’ classrooms (each class had between 20 to 25 students), conducted by a trained psychologist in collaboration with one masters-level psychology student. The sessions followed an interactive methodology using group dynamics, music, and videos which were adapted to the target group. Each session had specific goals. The first session included: (1) presentation of the project; (2) establishment of group rules; (3) exploration of students’ knowledge and beliefs about physical and mental health and illness; (4) exploration of the signs of mental health problems and their impact; (5) identification of risk factors for mental health; (6) identification of symptoms and signs of five mental disorders (depressive disorder, generalized anxiety disorder, anorexia, schizophrenia, and substance-related disorder); and (7) promotion of nonstigmatized behaviors towards mental disorders, addressing the social inclusion of people with mental health disorders. The second session aimed to: (1) explore inadequate beliefs related to mental disorders; (2) raise students’ awareness of mental health problems and their impact; (3) identify formal and informal help-seeking options; (4) promote first aid skills towards people with mental health problems; and (5) address self-help strategies and explore mental health promoting behaviors. The program was previously designed and tested with the target group, through the development of a pilot study, in order to identify participants’ needs, and to guarantee message (e.g., “wording”) and methodology accuracy. This pilot study, developed with students aged 12–14 years old, included two stages: (1) focus groups; (2) pilot-intervention with impact evaluation (pre-post intervention assessment) [33]. Information about the program and its manual can be found at http://www.fep.porto.ucp.pt/pt/AbrirEspacoSaudeMental?msite=12.

### 2.3. Measures

The MHLq—Mental Health Literacy questionnaire—was used to evaluate the effectiveness of the mental health promotion program [32].

The MHLq consists of two sections; in the first section sociodemographic data are collected, namely the date of birth, gender, year of schooling, and area of residence of the young person, and the professions and professional status of the parents. In this section, three questions are also given concerning knowledge of someone with a mental disorder, identification of the mental health disorder/problem, and relationship with the identified person.

The second section evaluates mental health literacy and consists of 33 multiple-choice items on a five-point Likert scale (from 1 = strongly disagree to 5 = strongly agree), organised in three dimensions: (1) Knowledge/Stereotypes (18 items); (2) First Aid Skills and Help Seeking (10 items); and (3) Self-Help Strategies (5 items).

The questionnaire assesses: (1) knowledge about mental health issues, including general characteristics of mental health problems, prevalence, signs and symptoms, and risk factors for mental disorders, as well as protective factors/mental health promoters; (2) knowledge of three specific mental disorders—depression, anxiety, and schizophrenia; (3) stereotypes associated with mental disorders; and (4) behavioral intentions (predisposition to help, behavioral promoters of mental health/self-help strategies, behaviors promoting the seeking of formal and/or informal help). At the follow-up stage three questions were added to the questionnaire in order to confirm participation in the intervention sessions and to measure satisfaction with the intervention, as well as request suggestions for improvement.

Higher values in all dimensions and for the total MHLq score correspond to higher levels of mental health literacy.

## 3. Procedures

### 3.1. Data Collection

The MHLq was applied one week before (pre-test), one week after (post-test) and six months after (follow-up) the intervention. It was carried out in a classroom setting by one of the researchers from the project team for “Finding Space for Mental Health”.

This study was approved by the Portuguese Data Protection Authority (ID 11098/2011) and by the Portuguese Ministry of Education (ID 0128800003).

Informed consent was given by participants and by the students’ caregivers, prior to their inclusion in the project. In order to pair participants in the three assessment moments, students were asked to provide five digits from their cell phone number (4th, 5th, 7th, 8th, and 9th digits).

After the follow-up assessment, students from the control group had the opportunity to attend the intervention program.

### 3.2. Analytic Plan

Continuous variables are described by the mean and standard deviation.

To evaluate the effectiveness of the intervention over the three moments in time (pre, post, follow-up), multivariate models (GEE, or generalized estimating equations) were applied, with the identity as a function of connection, i.e., a linear evolution was assumed. GEE is a method that allows the analysis of repeated or longitudinal measurements, taking into account that the measurements in the same individual over time are correlated. GEE provides many benefits, namely (1) accounts for within-subject/within-cluster correlations; (2) allows for time-varying covariate; (3) allows for irregularly timed or mistimed measurements; (4) provides consistent (i.e., asymptotically unbiased) parameter and standard error associated with the covariates of the model even when the correct correlation structure was not pick; (5) fits marginal models; (6) normality assumptions are not required; and (7) allows missing data [34,35]. As covariables (variables for adjustment), the pre-test scores and the variables defined in the study were used as possible confounding factors (pre-test score, gender, school year, type of school and knowledge of anyone with a mental health problem).

In this study, sum scores for the MHLq dimensions and global score were calculated. In all hypothesis tests, a level of significance of *α* = 5% was used. The analysis was performed using the statistical analysis program SPSS^®^ v.20.0 (Statistical Package for the Social Sciences, Armonk, NY, USA).

## 4. Results

The presentation of the results is divided in two sections: (1) presentation of the descriptive statistics of the MHLq for the experimental and control group, at the three times of evaluation; and (2) presentation of the results regarding the effectiveness of the intervention and the impact of the sociodemographic variables on the results of the intervention.

### 4.1. Descriptive Statistics on the Overall Score and Scores on the Dimensions of Mental Health Literacy

Table 2 shows the means and standard deviations of the experimental and control groups, at the three evaluation times—pre, post and follow-up—in relation to the global score and the dimensions of mental health literacy evaluated by the MHLq.

Differences between groups in the pre-test were studied in relation to the global score and MHLq dimensions. The only significant differences were found in the dimension “Self-Help Strategies”, in which the control group had higher initial values than the experimental group (*p* < 0.001).

### 4.2. Effectiveness of the Intervention and Impact of Sociodemographic Variables

Table 3 shows the results of the GEE models used to evaluate the effectiveness of the intervention, based on the data collected at the three evaluation times (pre, post and follow-up), for each of the dimensions and the global score of the MHLq. The results concerning the differential impact of sociodemographic variables on the outcome of the intervention are also presented, using multivariate models in which the coefficients presented are adjusted to all variables.

Participants in the experimental group had, on average, significantly higher gains compared to the control group, both in the global score (*β* = 7.707; 95% CI = 6.069; 9.345) and in all MHLq dimensions (Knowledge/Stereotypes *β* = 5.693; 95% CI = 4.682; 6.704; First Aid Skills and Help Seeking *β* = 0.744; 95% CI = 0.133; 1.356; Self-Help Strategies *β* = 1.236; 95% CI = 0.848; 1.624), demonstrating the effectiveness of the program through to follow-up.

In terms of the differential impact of the sociodemographic variables on the global score, it was observed that the participants who did not know someone with a mental health problem obtained lower gains than those who reported knowing someone with a mental health problem (*β* = −1.992; 95% CI = −3.949; −0.035).

Regarding the dimension of Knowledge/Stereotypes, it was observed that the students attending the ninth year had, on average, higher gains than students attending the seventh year (*β* = 1.719; 95% CI = 0.404; 3.034) and the participants who reported not knowing people with mental health problems showed, on average, gains that were significantly lower than those of the participants who reported knowing someone with these types of problems (*β* = −1.699; 95% CI = −2.935; −0.463).

With regards to the First Aid Skills and Help Seeking dimension, the results indicated that female participants showed, on average, gains that were significantly higher than the male participants (*β* = 0.777; 95% CI = 0.146; 1.407), and those enrolled in public schools showed, on average, gains that were significantly lower than the students from state-funded schools (*β* = −0.769; 95% CI = −1.462; −0.076).

Finally, on the Self-Help Strategies dimension, it was verified that the participants attending the ninth year obtained, on average, significantly higher gains than the participants of the seventh year group (*β* = 0.610; 95% CI = 0.115; 1.104).

## 5. Discussion

### 5.1. Effectiveness of Intervention

The present study involved 543 participants and aimed to evaluate the effectiveness of an intervention program to promote mental health literacy with youngsters from 12–14 years of age in a school context, as well as to explore the impact of sociodemographic variables on the intervention results.

GEE analysis informed us about the effectiveness of the intervention. As hypothesized, the participants of the EG had, on average, significantly higher values in the global score and in all dimensions of the MHLq compared to the CG, considering the evolution throughout the three evaluation times (pre, post, and follow-up). These results suggest that the intervention was effective, similar to the results found from other programs focused on promoting mental health literacy (e.g., [29,36]).

More specifically, there was a significant increase in Knowledge/Decrease of Stereotypes (Dimension 1). The literature has previously revealed the usefulness of short interventions to increase knowledge about mental health problems in young people (e.g., [11,29]).

As for the results obtained in Dimension 2—First Aid Skills and Help Seeking—the young people who benefited from the intervention were more likely to seek appropriate help for mental health problems, as well as to help others who present with them, and these results are also in line with the literature (e.g., [13]).

For Dimension 3—Self-Help Strategies—there was a statistically significant increase in the knowledge of self-help strategies in the EG, as reported in similar studies (e.g., [29]). These results can be explained by a positive view of the use of self-help strategies in young people and the general population, increasing the chance that people will show an interest in learning new forms of self-help as a way of coping with adversity arising from mental health problems like anxiety [4].

### 5.2. Exploration of the Impact of Sociodemographic Variables on Intervention Effectiveness

With regards to the hypothesized differences in gender, they were only statistically significant in the demand for First Aid Skills and Help Seeking. These results are in line with what is suggested in the literature, which indicates that in comparison to girls, boys show less intention to seek help and are less likely to help someone in need [25,26].

However, although the literature indicates that the intervention tends to have a greater impact for girls (e.g., [27,29]), in the present study there was a positive impact for both genders, in the global score and in the dimensions of Knowledge/Stereotypes and Self-Help Strategies.

On the variable of type of school, young people who attended public schools had, on average, significantly lower gains in the First Aid Skills and Help Seeking dimensions than those attending state-funded schools.

State-funded and public schools seem to be associated with lower and higher socioeconomic status (SES) [37], respectively. Previous research showed an influence of SES on MHL, with people with low SES tending to know less about the symptoms and prevalence of different mental health problems [38,39]. Preliminary results from the “Finding Space for Mental Health” project indicated that students from state-funded schools presented initially with lower levels of knowledge related to mental health problems, when compared to students from public schools [40]. As far as we know, no studies have focused on the influence of this variable on the impact of MHL promotion interventions. Future mental health promotion interventions and research on their effectiveness should consider school context specificities.

Regarding the variable of year of schooling, the oldest year group (ninth year) presented gains, on average, that were significantly higher in the dimension of Knowledge/Stereotypes and in the Self-Help Strategies dimension when compared to the younger youths (seventh grade). This result is in line with our previous research [32], where differences in MHL between 14–17 year old and 11–13 year old participants were found, with older students demonstrating higher scores in the Knowledge/Stereotypes and Self-Help Strategies dimensions, and the MHLq global score. Although this project is focused on a narrow age-span (12–14 year-old), it is interesting to notice age differences in the impact of the intervention concerning specific dimensions of MHL, which might be attributable to the rapid cognitive and socioemotional developmental changes in adolescence. This points to the importance of adjusting intervention strategies focused on the different dimensions of MHL according to the age and developmental stage of participants.

Finally, concerning the hypothesis regarding the impact of previous knowledge of someone with a mental health problem on the effectiveness of the intervention, young people who reported knowing people with a mental health problem had, on average, significantly higher gains than those who did not know someone with a mental health problem, on the Global Score and on the Knowledge/Stereotypes dimension. Previous research (e.g., [30,31,32]) showed higher levels of MHL in people who indicated knowing someone with a mental health problem. It is possible that young people who have already had contact with people with mental health problems are more willing to understand and discuss informative content on this subject and, consequently, benefit more from interventions of this type. The design of future interventions should take into account previous level of contact with mental health problems amongst those for whom the intervention is designed.

In future studies, some of the limitations of the present study may be addressed, namely those related to the lack of information concerning some variables that could also impact the effectiveness of the intervention, such as students’ socioemotional developmental characteristics, family SES and other parental variables, as well as identification of possible health-related programs developed in schools they attend. Furthermore, new research should explore the impact of the intervention program in specific groups of students, such as adolescents at risk of developing mental health problems, assessing their help-seeking behavior as a potential outcome. A larger dissemination of this program through the integration of its content into school curricula would also be an interesting research subject, already been studied by some authors [41]. Finally, considering the ubiquity of technology in young peoples’ lives, it is relevant to test the differential effectiveness of this intervention program, comparing its current delivery model to an updated model that includes technologically-mediated communication with the participants between sessions.

## 6. Conclusions

This study evaluated a school-based intervention program at three moments in time—pre-intervention, post-intervention and follow-up (six months after the intervention), using an experimental design that utilized assessment instruments adapted to the population, as recommended in the literature [22,42].

The results presented in this study should contribute to the broader discussion of how the promotion of mental health literacy in young people needs to be addressed. There is an urgent need to implement interventions that demonstrate positive impacts in the promotion of mental health literacy with young people—specifically in the increase of knowledge and reduction of stereotypes related to mental health problems, promotion of positive attitudes towards first aid skills and help-seeking behaviors, and development of self-help strategies.

Differences were observed in the gains from the intervention due to sociodemographic variables—gender, schooling year, previous contact with mental health problems, and school type (public vs. state funded).

The results of the study reinforce the usefulness of the most current and comprehensive version of the MHL concept, (e.g., not focusing exclusively on increasing knowledge about a particular disorder), as well as the need to take into account developmental and contextual variables in MHL promotion programs.

## Figures and Tables

**Table 1:** Differences between the experimental group and control group regarding sociodemographic variables (pre-test).

	Control Group		Experimental Group		*p*-Value ^1^
	*n*	(%)	*n*	(%)	
Gender					0.731
Male	148	(53%)	130	(51%)	
Female	133	(47%)	124	(49%)	
School Year					0.936
7th year	74	(26%)	64	(25%)	
8th year	105	(37%)	98	(38%)	
9th year	105	(37%)	97	(37%)	
Type of Education					<0.001
State-Funded School	245	(86%)	154	(59%)	
Public School	39	(14%)	105	(41%)	
Parents’ professional situation					0.770
Employed	221	(86%)	240	(85%)	
Unemployed	36	(14%)	42	(15%)	
Knowledge of anyone with a mental health problem					0.317
Yes	114	(57%)	122	(62%)	
No	86	(43%)	75	(38%)	
Degree of Proximity					0.617
Relative	49	(40%)	54	(43%)	
Friend	45	(37%)	53	(42%)	
Him/herself	5	(4%)	3	(2%)	
Other	23	(19%)	16	(13%)	

^1^ Chi-square test.

**Table 2:** Descriptive statistics concerning the global score and dimensions of mental health literacy at the three evaluation times—pre, post and follow-up.

	Global Score		Knowledge/Stereotypes		First Aid Skills and Help Seeking		Self-Help Strategies	
	Control	Experimental	Control	Experimental	Control	Experimental	Control	Experimental
Pre								
Mean (sd)	130.86 (13.23)	129.75 (11.15)	69.39 (8.22)	69.00 (7.17)	41.60 (5.41)	41.58 (4.71)	19.94 (3.14)	19.17 (3.11)
n	284	259	284	259	284	259	283	259
Post								
Mean (sd)	130.58 (13.08)	140.10 (12.58)	69.02 (8.23)	75.91 (8.13)	41.64 (5.34)	42.74 (4.68)	19.91 (3.16)	21.44 (2.70)
n	263	239	263	239	263	239	263	239
Follow up								
Mean (sd)	134.77 (11.02)	137.76 (11.88)	72.19 (6.82)	75.36 (7.25)	41.81 (5.01)	41.52 (4.96)	20.77 (2.70)	20.89 (2.86)
*n*	176	211	176	211	176	211	176	211

**Table 3:** Study of intervention effectiveness and the differential impact of sociodemographic variables based on Generalized Estimating Equations using repeated measurements (pre, post and follow-up) adjusted for some variables (Pre-test scores, Gender, School Year, Type of School and knowledge of anyone with a mental health problem) for global score and dimensions of mental health literacy.

		Global Score (*n* = 508)			Knowledge/Stereotypes (*n* = 508)			First Aid Skills and Help Seeking (*n* = 508)			Self-Help Strategies (*n* = 507)		
		*β*	95% CI	*p*	*β*	95% CI	*p*	*β*	95% CI	*p*	*β*	95% CI	*p*
Group	Control	Ref	-		Ref	-		Ref	-		Ref	-	
	Experimental	7.707	6.069; 9.345	<0.001	5.693	4.682; 6.704	<0.001	0.744	0.133; 1.356	0.017	1.236	0.848; 1.624	<0.001
Pre-Test score		0.523	0.442; 0.604	<0.001	0.505	0.420; 0.590	<0.001	0.589	0.523; 0.654	<0.001	0.420	0.347; 0.492	<0.001
Gender	Male	Ref			Ref			Ref			Ref		
	Female	1.429	−0.199; 3.057	0.085	0.750	−0.267; 1.767	0.149	0.777	0.146; 1.407	0.016	−0.168	−0.543; 0.208	0.382
School Year	7th year	Ref			Ref			Ref			Ref		
	8th year	0.778	−1.344; 2.900	0.472	1.086	−0.251; 2.423	0.110	0.086	−0.722; 0.894	0.835	−0.313	−0.821; 0.195	0.227
	9th year	1.500	−0.504; 3.505	0.142	1.719	0.404; 3.034	0.010	−0.499	−1.296; 0.297	0.219	0.610	0.115; 1.104	0.016
Type of School	State-Funded School	Ref			Ref			Ref			Ref		
	Public School	−1.612	−3.487; 0.263	0.092	−0.724	−1.928; 0.480	0.239	−0.769	−1.462; −0.076	0.030	−0.124	−0.583; 0.335	0.595
Knowledge of anyone with mental health problem	Yes	Ref			Ref			Ref			Ref		
	No	−1.992	−3.949; −0.035	0.046	−1.699	−2.935; −0.463	0.007	−0.214	−0.943; 0.514	0.564	−0.108	−0.538; 0.321	0.621
	Don’t Know	−1.467	−3.444; 0.510	0.146	−1.129	−2.384; 0.126	0.078	−0.228	−0.970; 0.515	0.548	−0.114	−0.582; 0.354	0.633

Type of Model: scale response: linear; Ref—Category of reference; 95% CI—95% confidence interval; Reading example: *β* = 7.707—an expected difference in global score comparing experimental to control group of the same gender, school year, type pf school and knowledge of anyone with mental health problem.
